# Improving the quality of chemical language model outcomes with atom-in-SMILES tokenization

**DOI:** 10.1186/s13321-023-00725-9

**Published:** 2023-05-29

**Authors:** Umit V. Ucak, Islambek Ashyrmamatov, Juyong Lee

**Affiliations:** 1 Department of Molecular Medicine and Biopharmaceutical Sciences, Graduate School of Convergence Science and Technology, Seoul National University, Seoul, Republic of Korea; 2 College of Pharmacy, Seoul National University, Seoul, Republic of Korea; 3 Research Institute of Pharmaceutical Science, Seoul National University, Seoul, Republic of Korea

**Keywords:** Atom-in-SMILES, Tokenization, Repetition, Chemical language processing

## Abstract

Tokenization is an important preprocessing step in natural language processing that may have a significant influence on prediction quality. This research showed that the traditional SMILES tokenization has a certain limitation that results in tokens failing to reflect the true nature of molecules. To address this issue, we developed the atom-in-SMILES tokenization scheme that eliminates ambiguities in the generic nature of SMILES tokens. Our results in multiple chemical translation and molecular property prediction tasks demonstrate that proper tokenization has a significant impact on prediction quality. In terms of prediction accuracy and token degeneration, atom-in-SMILES is more effective method in generating higher-quality SMILES sequences from AI-based chemical models compared to other tokenization and representation schemes. We investigated the degrees of token degeneration of various schemes and analyzed their adverse effects on prediction quality. Additionally, token-level repetitions were quantified, and generated examples were incorporated for qualitative examination. We believe that the atom-in-SMILES tokenization has a great potential to be adopted by broad related scientific communities, as it provides chemically accurate, tailor-made tokens for molecular property prediction, chemical translation, and molecular generative models.

## Introduction

Tokenization is an essential preprocessing step for sequential data to train and use natural language processing (NLP) models. However, insufficient attention has been devoted to its effects on chemical applications. Tokenization can significantly influence prediction quality within the framework of text generation [1]. In the field of chemistry, it covers processes used to split linear molecular representations into their constituent elements. As linear molecular representations are algorithmic abstractions, their partitioning can alter the perception of molecules. Herein, tokenization refers to any logical partitioning of molecular structures based on SMILES strings.

In general, a molecule can be perceived as an inherent whole, owing to the internal relationships among its atomic components. Simplified Molecular Input Line Entry System (SMILES) strings [2], the most commonly used molecular representation, are also defined to be meaningful as a whole. They represent molecular objects, which are rigid bodies and completely different from their constituent atoms. Notably, any sensible partitioning of a molecule will produce meaningful fragments. However, to some extent, the abstraction level of the process will get tangled because considering atoms in a molecule is not a “realistic” approach. Likewise, SMILES tokens are merely sensible linear cuts of a string with reduced dimensionality.

A typical tokenization of SMILES is performed atom-wise, i.e., character-wise. However, the SMILES representation consists of a small number of distinct characters including atomic symbols, integers for ring closure, and special symbols for bonds and chirality. In other words, all atoms with the same atomic number are represented identically. However, the characteristics of each atom even with the same atomic number may differ significantly based on its environment. This statement is similar to the concept of atoms in molecules (AIM) [3], which describes the nature of molecules based on the electron density distribution attracted by each atom. Thus, the conventional atom-wise tokenization of SMILES may be too abstract and chemically inaccurate and it may obscure the learning process of a model and the understanding of the results.

Herein, we point out an analogy between the natural (spoken) language and constructed language of chemistry (see Table 1). The analogy provides a motivational ground for the use of NLP methods in chemistry problems  [4]. The intended analogy relies on the part-whole relationship  [5, 6] and suggests that molecular substructures (typically composed of several atoms) can be considered as chemical “words” for the linguistic treatment of chemical language. However, in practice, chemical words often become the tokens of SMILES that consists of atomic symbols and characters representing topological characteristics, such as ring-closure or branches, which do not correspond to physical atoms. In this context, atoms are present in molecules by neglecting an essential aspect of the chemical reality. In the following paragraph, we analyze the token characteristics of sentences and SMILES strings for insight into the influence of the latter format on the translation mechanic.

According to the sentence length distribution of various language corpora, a well-written sentence contains 15–20 words on average [7]. The average sequence length of a SMILES string is typically three times longer than a natural language, whereas the token space is at least 1000 times smaller than any developed language [8]. This is a consequence of repetitive tokens observed in SMILES strings. The most distinguishing feature of SMILES representation is the token repeat, which causes atoms of molecules to be indistinguishable in the token space. The repetitive nature of SMILES syntax adds to the more general issue of neural machine translation (NMT) decoders, yielding degenerative outcomes [9, 10].

Token order is another aspect of this comparison. Although the order of words in a sentence can be altered to enhance tone, meaning, or fluency, this cannot be applied to molecules. In fact, a single molecule can equally be represented by hundreds of SMILES enumerations depending on its topology (more if branches and cyclic fragments exist)  [11]. Canonical SMILES refers to one of those many allowed permutations obtained by a unique and consistent atom numbering. In essence, while words tend to retain their semantic significance as they transition from isolated to contextual settings, with only minor semantic shifts that may occur over time, the same does not hold true for atoms within the realm of chemistry. For example, the atomic symbols within a SMILES string are treated equivalently to those in isolation, signifying that the chemical significance of these symbols is upheld throughout the tokenization process. Thus, tokens such as carbon (C) may appear identical in different molecules despite their actual differences in chemical composition. However, it is important to distinguish between atom-in-SMILES (AIS, analogous to AIM) and corresponding tokens, as atoms lose their identities when they form molecules.

Inspired by the aforementioned comparative analysis outlined in Table  1, we develop a tokenization framework by introducing environmental information and show that it corroborates the chemical viewpoint. In recent decades, various methods have been developed to enhance or extend the SMILES language. Few of these methods include BigSMILES for describing macromolecules [12], CurlySMILES for supramolecular structures and nanodevices [13], CXSMILES for storing the special features of molecules [14], OpenSMILES specification for specifying the stereochemistry and chirality [15], DeepSMILES and SELFIES for machine learning applications [16, 17], and canonicalization algorithms [18, 19]. The aforementioned approaches effectively solve particular problems originating from the internal structure of SMILES. In our approach, we do not treat syntactic problems; rather, we redefine SMILES tokens by introducing environmental information. To consider local chemical environments, atom environments (AEs) are used, which are circular atom-centered topological molecular fragments created with predefined radii of covalent bonds. Hence, our approach entails utilizing AEs to produce environment-aware atomic tokens analogous to atom-in-molecules. We term this custom tokenization scheme Atom-in-SMILES, AIS.

**Table 1:** Comparison of the important aspects of natural and chemical languages within the NLP framework

Aspects	Natural language	SMILES language
Sequence length	15-20 words	$\sim$ 3 times higher
Token space	>100K	$\sim$ 1000 times smaller
Token order	Tone, meaning, fluency	${}_n C_{2}$ alternatives^*^
Meaning-wise	isolation $\equiv$ context	isolation $\equiv$ context

*practically less due to the rules of chemistry

The AIS tokenization integrates key aspects of SMILES and AEs [20]. The proposed approach accommodates all relevant information for a seamless bidirectional transformation between the two representations, to ensure practical implementation. We demonstrate that the AIS scheme performs better in translating between equivalent string representations of molecules using an exceptionally challenging dataset. It was also observed that training with AIS tokenization leads to more accurate models for single-step retrosynthetic pathway prediction, and molecular property prediction tasks. We also evaluated prediction qualities by comparing AIS tokenization with the existing schemes: canonical SMILES-based tokens, atom-wise and SMILES pair encoding (SmilesPE) [21], SELFIES, and DeepSMILES tokens. We show that AIS tokenization reflects the true chemical context, delivers better performance, and reduces token degeneration by 10%.

## Implementation

**Fig. 1:**
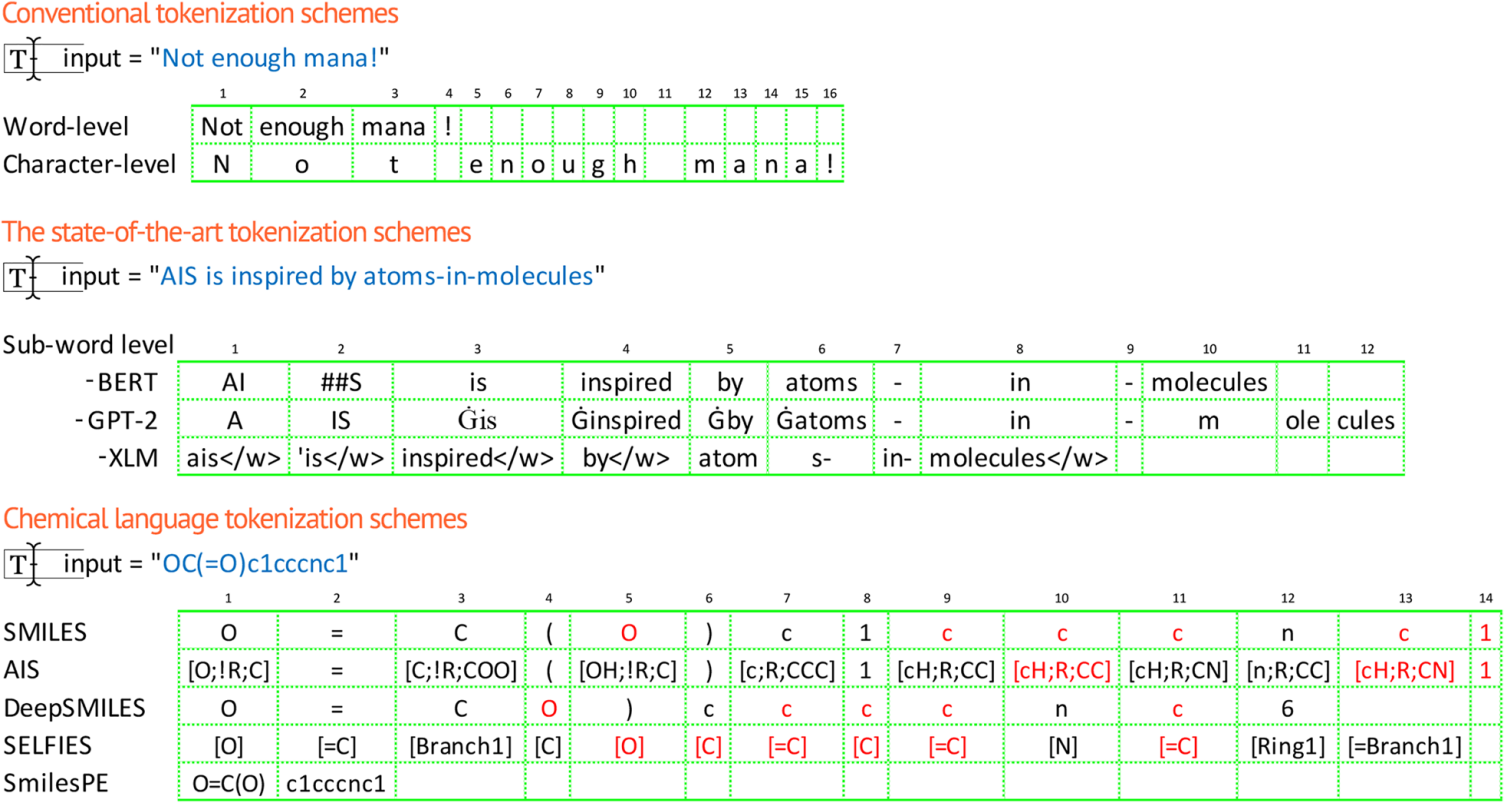
Comparison of conventional and modern tokenization schemes in NLP and the tokenization methods in the chemical language domain

Advanced tokenization schemes have emerged as a result of the evolution of natural language processing. Figure 1 shows that state-of-the-art tokenization schemes, like BERT [22], GPT-2 [23], and XLM [24], divide words into sub-words to capture contextual relationships between them while conventional tokenization schemes used to break down sentences into words or characters. In the field of cheminformatics, atom-wise tokenization of SMILES is primarily used for training chemical language models. In addition to atom-wise SMILES tokenization, new molecular representations have been introduced such as SELFIES and DeepSMILES, and specialized tokenization schemes like SmilesPE imitating byte-pair encoding.

**Fig. 2:**
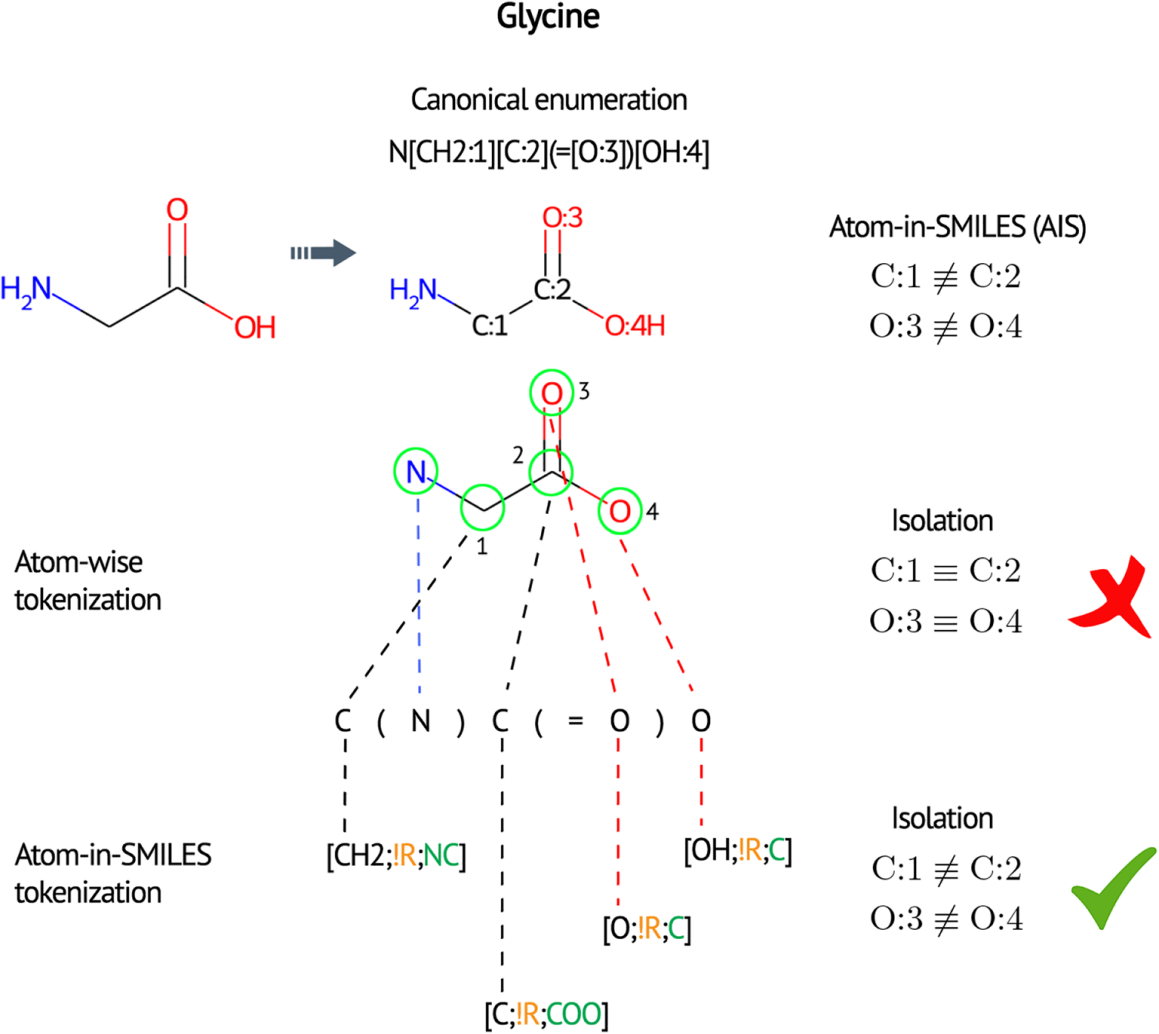
A toy example illustrating the major differences between AIS and conventional SMILES tokenizations. The formal description of AIS tokenization contains three primary elements, (i) central atom, (ii) ring information, and (iii) neighbor atoms information, interacting with the central atom. The formalism ties everything together within a square bracket separated by a semi-colon. The chirality information can be attached to the central atom, which is labeled with either *@* or *@* *@* suffixes. Aromaticity is reflected on the central atom with a lower case letter. Hydrogen atoms are explicitly specified on central atoms. The hybridization and bonding nature of organic elements can be easily deduced

In the token space generated by atom-wise SMILES tokenization, all atoms with the identical atomic numbers are indistinguishable. As a toy example, in a glycine molecule (Fig. 2) carbons are represented as two identical carbon atoms following tokenization. Oxygen atoms are also treated similarly. Hypothetical atomic constituents obtained by tokenization are often degenerated. This is an intrinsic feature of SMILES representation, which does not correspond to chemical reality.

We propose the AIS tokenization scheme that expresses. The most natural formulation of this proposition is as follows. Let $T_{1}$ and $T_{2}$ be the token spaces of SMILES and AIS, respectively, and $f:T_{1}\rightarrow T_{2}$ be a mapping, which is one-to-one and onto; then,

1 $$\begin{aligned} f(X)= {\left\{ \begin{array}{ll} \left. AE\right| _{X_\text{central}} &{}\text {if { X} is an atom} \\ X &{}\text {otherwise}. \end{array}\right. } \end{aligned}$$

For any SMILES string, the function *f* simply tweaks each atom by selecting it as the central atom of the corresponding atomic environments (AEs); otherwise, it is an identity operator from the token space. The chirality and aromaticity information of the central atom are preserved through the above-described mapping. Bond order and hybridization are the two intrinsic dimensions of AIS tokens. As *f* is invertible, SMILES strings can be fully recovered by the SMILES projection. The proposed algorithm for generating AIS tokenization can be described through the presented pseudo-code (Algorithm 1). The algorithm works by iterating over the atoms in a SMILES string and generates rich, environment-aware variants.

**Fig. 3:**
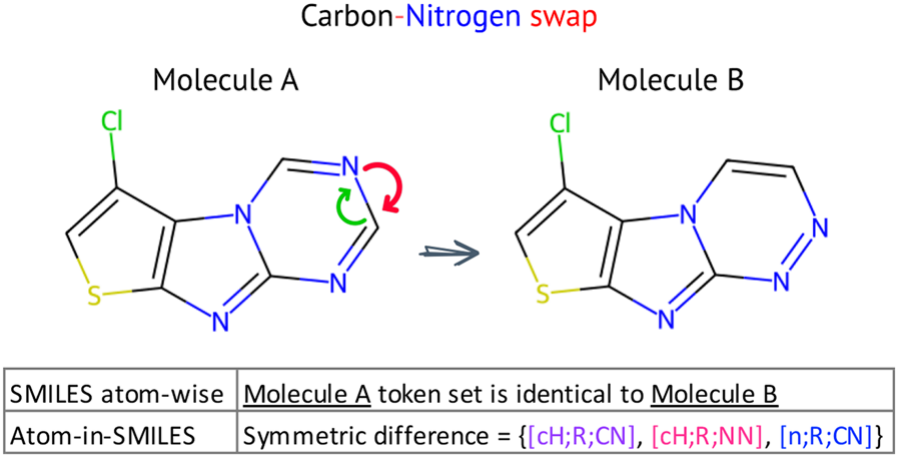
Token set comparison of two highly similar molecules. The molecules differ only in the position of a carbon and nitrogen atom in one of the rings

Introducing the neighboring atoms interacting with the central atom generates tokens with greater diversity. As shown in Fig.  2 carbon and oxygen atoms are well distinguished according to their local chemical environments ($\text {C:1} \not \equiv \text {C:2}$, $\text {O:3} \not \equiv \text {O:4}$). The token space stretches relative to the atom-wise tokenization. As another example, shown in Fig. 3, we can consider the tokens of the following aromatic molecules: Clc1csc2nc3ncncn3c12 and Clc1csc2nc3nnccn3c12. The atom-wise tokens of these molecules are identical. However, the set of the symmetric difference of AIS tokens has three members, [cH;R;CN], [cH;R;NN], and [n;R;CN], rendering the carbon-nitrogen swap recognizable.
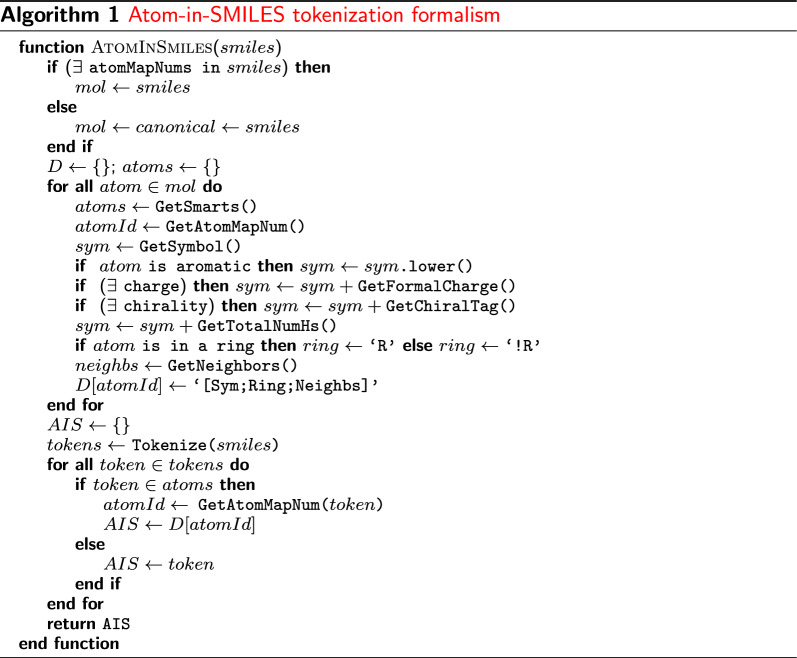


**Fig. 4:**
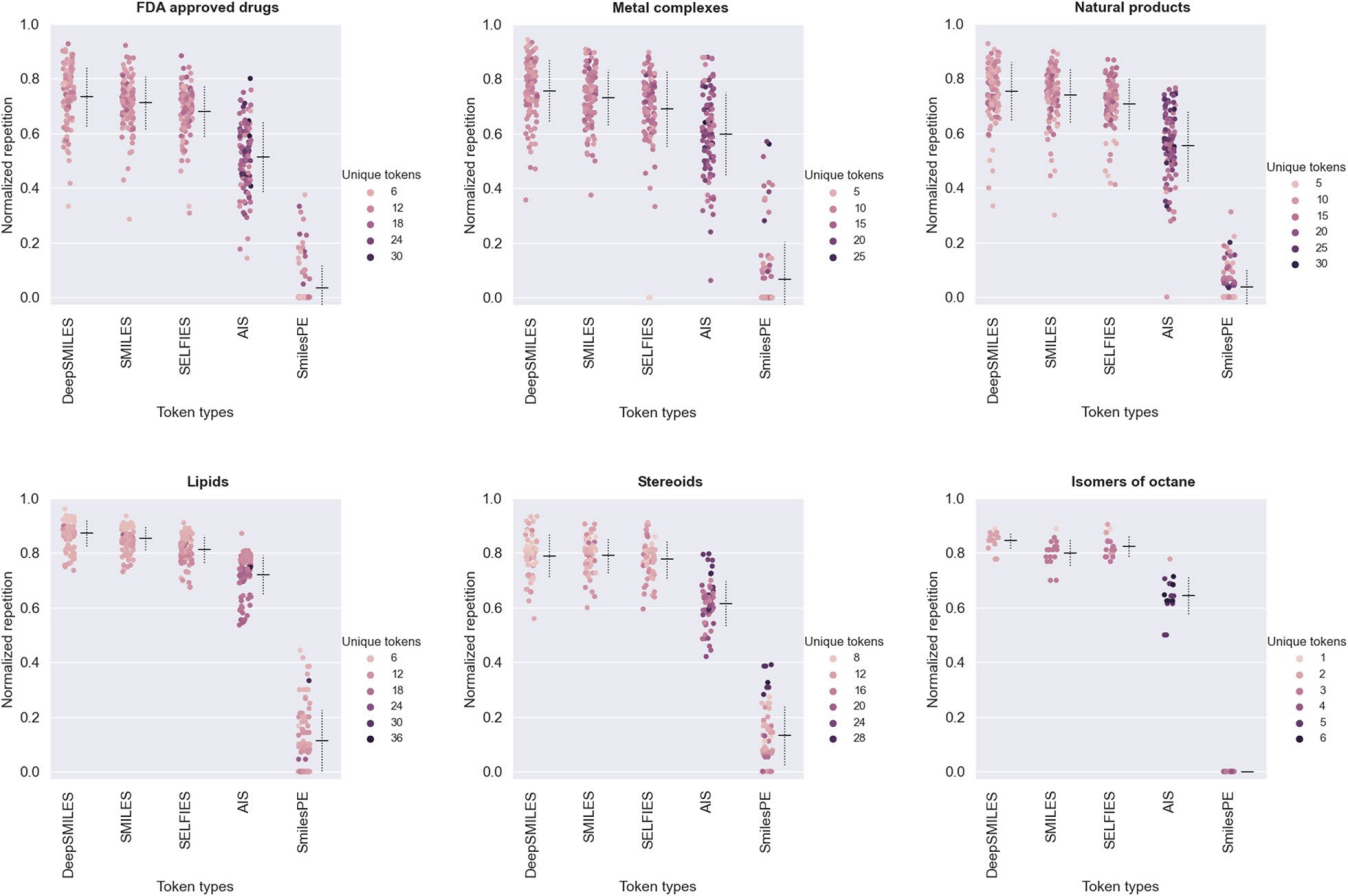
Comparison of expressiveness and normalized repetition rates across various molecular representations. Distributions showcasing the distinct characteristics of tokenization schemes on representative datasets, each designed to test different facets of molecular structures such as coordination compounds, ligands (metal complexes), ring structures and functional groups (steroids), long-chain formations (phospholipids, ionizable lipids), complex and diverse structures (natural products), small organic molecules (drugs), and configurational changes in molecular structure (octane isomers). Each dataset contains one hundred members, with the exception of steroids (59 members) and octane isomers (18 members). The mean values of normalized repetitions and deviations from the mean are visually represented as horizontal and dashed vertical lines, respectively, accompanying the distributions

Figure 4 provides insight into the inherent properties of various molecular representations, revealing their expressive power, token diversity, and chemical relevance. We evaluated the distributions of tokens and normalized repetition rates across a diverse set of molecular datasets with a wide range of structural complexities and configurational changes, such as coordination compounds and ligands (metal complexes from Crystallography Open Database [25]), ring structures and functional groups (steroids [26]), long-chain formations (phospholipids and ionizable lipids  [27]), complex and diverse structures (natural products [28]), small organic molecules (drugs [29]), and configurational changes in molecular structure (octane isomers). Single-token repetition can be easily quantified as $\text{ rep-l } = \sum ^{|s|}_{t=1} [s_t \in s_{t-w-1:t-1}]$, where $s$ and $|s|$ denote the prediction and token count respectively [10]. We kept the number of considered previous token $w$ sufficiently large (as large as the maximum sequence length). Normalized repetition rates, which measure the ratio of single-token repetitions to sequence length, is used to provide a meaningful measure of expressiveness. Lower repetition rates indicate more diverse and informative token sets that can alleviate the problem of degeneracy observed in model outcomes.

In Fig. 4, AIS tokens exhibit consistently lower repetition rates compared to SMILES, SELFIES, and DeepSMILES, indicating a higher level of expressiveness. This difference in expressive power is particularly evident in drugs, natural products, and steroid datasets. However, in expressing long chains, as in the case of lipids, all tokenization schemes struggle. One limitation of AIS is that it lacks the ability to distinguish environmentally similar substructures or those with a symmetry plane since it only considers nearest neighborhoods. The SmilesPE representation exhibits a low-lying distribution due to the relatively low number of pseudo-substructures with fewer or zero repetitions. It is worth noting that inherent repetitions in molecular representations can exacerbate the repetition problem observed in NLP model outcomes, highlighting the importance of diverse and informative token sets.

## Results and discussion

We tested the AIS tokenization on three challenging tasks: (i) input–output equivalent mapping (SMILES canonicalization), (ii) single-step retrosynthetic prediction, and iii) molecular property prediction. For the first two functionality tests, we utilized NMT framework that translates sequences from the source to the target domain with the most promising attention-based transformer encoder-decoder architecture [30, 31]. We trained our models for 200,000 steps with the Adam optimizer, negative log-likelihood loss, and cyclic learning rate scheduler. For these tasks, we report the percentage of exact prediction.

### Input–output equivalent mapping

First, we tested how the learning efficiency of an NMT model is affected by the choice of the tokenization scheme, on the task of converting non-canonical SMILES strings into their canonical form. For rigorous test, we generated extremely confusing datasets consisting of many similar strings. To generate the datasets, we used the predefined subsets of the GDB-13 [32] database that contains drug-like molecules with up to 13 heavy atoms which consist of C, N, O, S, and Cl. The subsets were generated by applying cumulative pre-defined constraints [33, 34], which were named as follows: a: No cyclic HetHet Bond; b: No acyclic HetHet Bond; c: Stable FG; d: No cyclic C=C and C:C bonds; e: No acyclic C=C and C:C bonds; f: No small rings; g: Fragment-like, and h: Scaffold-like. Our training dataset consisted of one million randomly sampled molecules taken from the GDB-13, combined with 150K randomly sampled from the most stringent GDB-13 subset abcdefgh. We augmented the subset at different levels ($\times$10, $\times$30, and $\times$50) to make the training set more confusing. This approach resulted in training datasets with a high degree of similarity between the input (non-canonical instances) and output (only canonical enumerations) SMILES strings, making it difficult to discern variations.

**Fig. 5:**
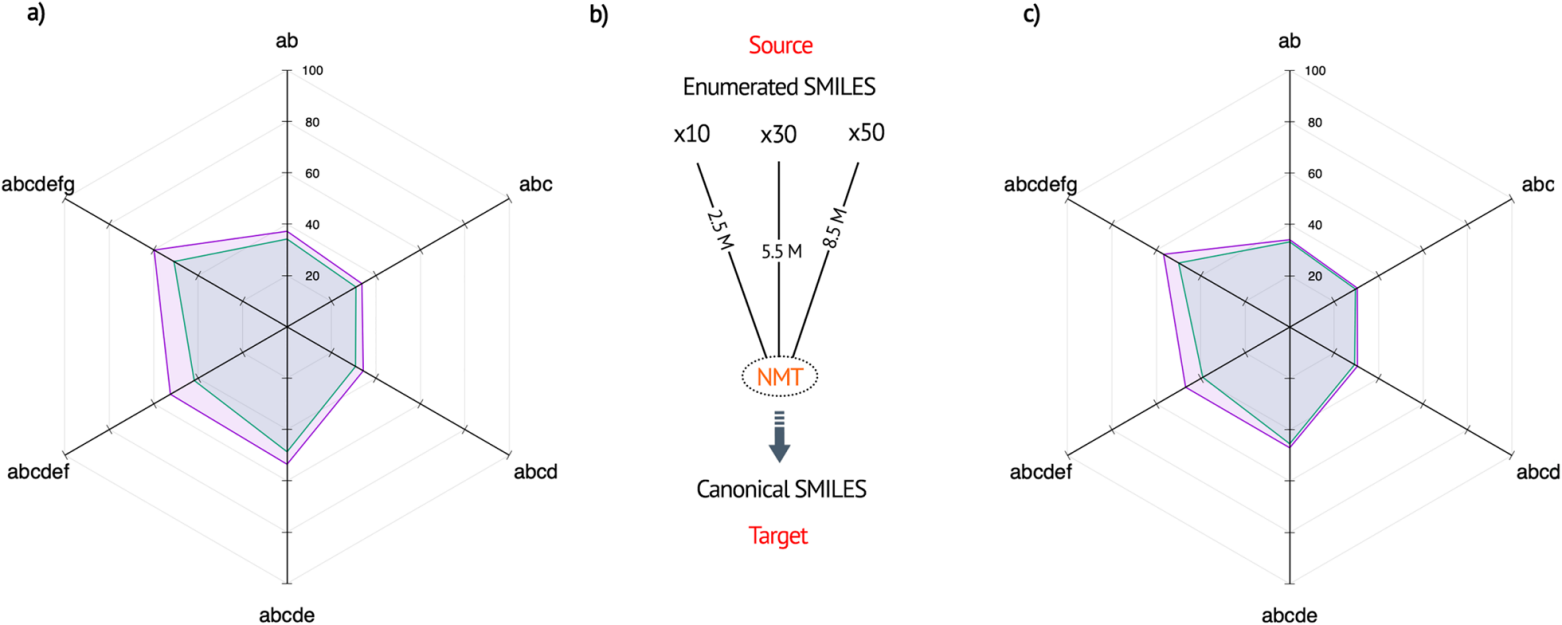
Performance of atom-wise (blue) and atom-in-SMILES (purple) tokenization schemes tested on various restricted GDB-13 test sets [33]. **a** Test results of $\times$10 augmented training set. **b** Model overview. **c** Test results of $\times$50 augmented training set. The training is conducted with one million randomly sampled molecules taken from the GDB-13, combined with 150K randomly sampled subset of the strictest cumulative abcdefgh data, which we augmented at different levels ($\times$10, $\times$30, and $\times$50)

We quantified the performance on large (20K) GDB-13 disjoint test sets of varying constraints (see Table 2). To highlight the benefits of our token design over SMILES tokenization, we utilized an approach shown in Fig. 5b. Table 2 and Fig. 5a, c demonstrate the limitations of SMILES tokenization and the characteristics of our tokens. The atom-in-SMILES scheme outperformed the SMILES atom-wise scheme on all subsets and augmentation levels, with increasing performance gaps for more restrictive subsets (more similar). The highest prediction accuracy of 59.9% (x10) and 56.8% (x50) was achieved on the subset abcdefg, compared to 50.9% (x10) and 50.0% (x50) for the atom-wise scheme.

In our experiments, we observed that the added complexity by data augmentation resulted in a degradation of performance, different from the typical degradation observed in overly complex models (overfitting) [35]. Atom-wise tokens struggled to handle the increasing complexity, resulting in a performance deficit of up to 10.7% on the abcdef subset. Notably, as the level of augmentation increased, the model’s token-level probabilities decreased. However, we found that the AIS tokenization, trained on a dataset of extremely similar molecules, was better equipped to handle this problem. The greater string similarity led to consistent improvements in predictive power, which we attribute to the richer and more expressive representation of AIS tokens.

**Table 2:** Performance of atom-wise and atom-in-SMILES tokenization schemes tested on various restricted GDB-13 test sets [33]

GDB-13 subsets [33] (cumulative)	Prediction accuracy (%)					
	Atom-wise			Atom-in-SMILES		
	x10	x30	x50	x10	x30	x50
ab	34.2	34.3	33.2	37.3	35.9	34.1
abc	31.0	30.8	29.6	33.7	32.1	30.4
abcd	30.8	30.4	29.2	34.3	32.3	30.5
abcde	48.7	47.6	45.5	53.6	50.0	47.0
abcdef	41.8	40.6	39.1	52.5	49.6	46.9
abcdefg	50.9	50.9	50.0	59.9	58.6	56.8

The training is conducted with one million randomly sampled molecules taken from the GDB-13, combined with 150K randomly sampled subset of the strictest cumulative abcdefgh data, which we augmented at different levels ($\times$10, $\times$30, and $\times$50)

### Single-step retrosynthesis and token degeneration

Retrosynthetic prediction is a challenging task in organic synthesis that involves breaking down a target molecule into precursor molecules using a set of reaction templates. This process helps chemists identify potential routes for synthesizing novel chemical structures. However, conventional template-based methods have limitations such as coverage and template generation issues  [36, 37], and can be computationally expensive [38]. Additionally, atoms have not been successfully mapped between products and reactants in these methods [39]. To address these challenges, we implemented a template-free, direct translational method to suggest reactant candidates, which is extremely similar to the concept proposed by several groups  [40–44]. These approaches can provide high-quality and complete recommendations without the need for hand-crafted templates [45] or pre-existing reaction databases [46, 47].

We adopted the open-source ca. USPTO-50K reaction benchmark dataset that is widely used for a single-step retrosynthesis prediction task. This dataset was a subset of a larger collection from the U.S. patent literature obtained with a text-mining approach  [48, 49]. As a preprocessing step, we removed sequences longer than 150 tokens. The prediction quality is assessed by top-1 accuracy, string match. Additionally, we reported Tanimoto exactness (with hashed Morgan Fingerprint radius of 3 and bit size of 2048 [50]) since the predicted structures might fail on the string match tests [51], but still can map to correct ground truth due to multiplicity of SMILES representation. To determine the effect of the tokenization on the prediction quality, we compared the performance of the AIS tokenization with two other SMILES-based tokenization schemes, namely, atom-wise and SmilesPE, and two molecular representations DeepSMILES and SELFIES.

**Table 3:** Degenerate repetition observed in various tasks from state-of-the-art large-scale language models

Task [model]	Context	[Repetition pattern] output
Sentence completion [GPT2] ref. [10]	**Prefix:** Lyrically the song has excerpts of different languages including French, Spanish,	[Single-word repetition] German, Italian, Portuguese, Spanish, **Portuguese**, Portuguese, Portuguese, Portuguese, Portuguese, Portuguese, Portuguese, Portuguese, Portuguese, .
Summarization [Transformer] ref. [52]	**Article**	[Phrase-level repetition] ...cristiano ronaldo’s best footballer whereas barcelona talisman lionel messi’s **best footballer **whereas barcelona’s best footballer whereas barcelona talisman lionel lee whereas’s best footballer’s. (Truncated)
Generation from an initial tag line [GPT-2] ref. [9]	**Tag:** So what’s new in my life? 09/11/18 - Just got back from vacation	[Sentence-level repetition] Just got back from vacation. 09/10/18 - **Just got back from vacation**. Just got back from vacation. 09/09/18 - Just got back from vacation. Just got back from vacation. 09/08/18 - Just got back from vacation. Just got back from vacation.
Product review generation [GPT-2] ref. [53]	**Initial context**	[Structural repetition] Great movie, although took a while to see at first it held my interest and kept me interested, plus i thought it was extremely good. also it was **very good**.
Protein sequence generation [ProtGPT-2] ref. [54]	**No context**	[Subsequential repetition] MSNDTPTHDPTPPAPAPAPE**PAPAPAPE**PAPAPAPEPAPAPAPEPAPAPAPEPAPAPAPEPAPAPAPEPAPAPAPEPAPAPAPEPAPAPAPEPAPAPAPE.
Molecule captioning [Transformer] ref. [55]	**SMILES:** CC[N+](CC)=C1C=CC2=N C3=C(OC2=C1)C=C(N)C(C) =C3	[Single-word repetition] the molecule is a deuterated compound that is **is** is is is an isotopologue of chloroform in which the four hydrogen atoms have been replaced by deuterium. (Truncated)

The examples contain single-word repetitions, phrase-level repetitions, sentence-level repetitions, structural repetitions where tokens may vary within a repeating phrase, and subsequential repetitions. The first repeated unit in each example is  emphasized in bold.

Repetition is a well-known issue in text generation models, where multiple tokens predict the same subsequent token with high probability [9, 56], leading to the generation of repetitive sequences. A sequence is said to have a repetition subsequence if and only if it contains at least two adjacent identical continuous subsequences [56]. Large-scale language models such as Transformer and GPT-2 have shown to exhibit this issue, resulting in a negative impact on the quality of generated text. The Table 3 demonstrates different types of token degeneration, including single-word repetition, phrase-level repetition, sentence-level repetition, structural repetition, and subsequential repetition. The examples are drawn from a range of NLP tasks, such as sentence completion, summarization, generation from an initial tag line, product review generation, protein sequence generation, and molecule captioning. This emphasizes the prevalence of token degeneration and highlights the importance of addressing this issue to ensure the generation of high-quality natural language text.

**Fig. 6:**
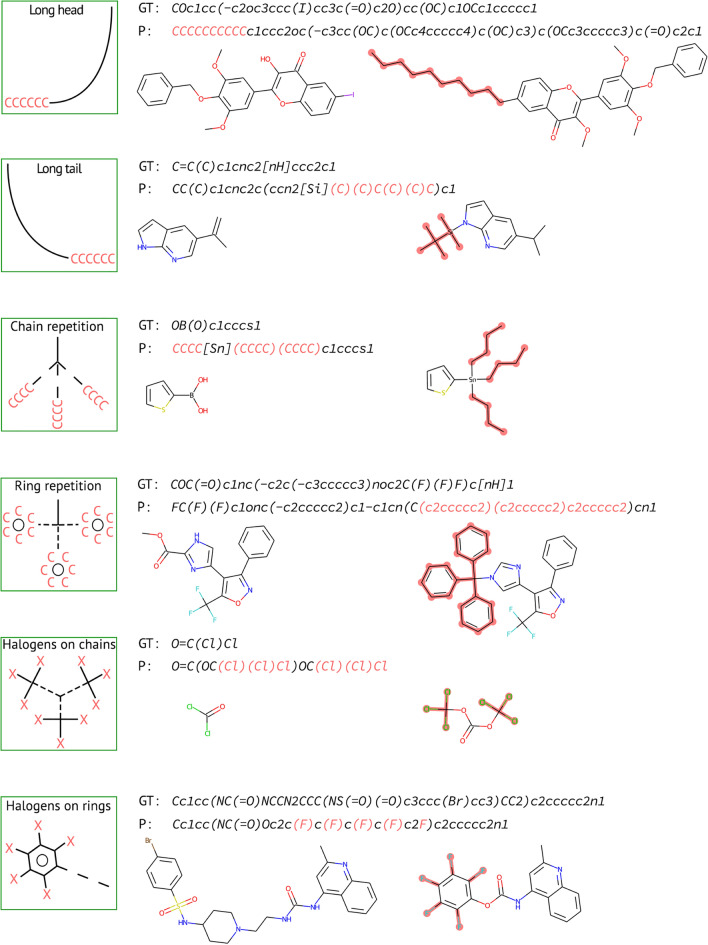
The most commonly occurring repetitive patterns in an NMT retrosynthetic framework. Copious repetitions are highlighted in SMILES and molecular drawings. GT and P refer to the ground truth and prediction, respectively

Herein, we observed that molecular prediction tasks are also susceptible to token repetition. With the careful examination of non-exact predictions, we were able to summarize the common forms of problematic outcomes. Figure 6 displays six typical examples of token repetition in SMILES predictions within an NMT retrosynthesis framework: long head and tail, repetitive rings and chains, and halogen repetitions on aliphatic and aromatic carbons. These outcomes are considered to be the most probable by the model and have a negative impact on the quality of predictions. The long head and tail result from the repeated addition of identical or similar substructures to a terminal, whereas repetitive rings and chains occur due to the repeated addition of the same substructure. The halogen repetitions on aliphatic and aromatic carbons occur when the model repeats the same halogen substitution on similar carbons. Understanding and addressing these problematic outcomes is crucial for the development of accurate molecular prediction models.

Methods for quantifying the propensity of subsequent repetition are adapted from the recent studies by Welleck [10] and Fu [56] on neural text degeneration. We focused more on token-level measure for repetition than sequence-level repetition [9] because NMT typically uses a maximum log-likelihood training objective that is concerned with optimizing next-token conditional distributions. We used a token-level measure for repetition, rep-l, that counts the single-token repeats appearing in the preceding tokens. As there are so many single-token repeats appear in the ground truth SMILES, we reported the number of predicted SMILES with repetition rate higher than the ground truth.

**Table 4:** Performance (top-1 accuracy) of various tokenization schemes on single-step retrosynthesis task and the number of predictions with token repetition

Tokenization schemes	$\text {rep-l}|_{P} - \text {rep-l}|_{GT} \ge 2$	Acc.(%) greedy	
		String exact	Tc exact
Atom-wise baseline [57]	–	42.00	–
Atom-wise (ref. [57] is reproduced)	801	42.05	44.72
SmilesPE (ref. [21])	821	19.82	22.74
SELFIES (ref. [17])	886	28.82	30.76
DeepSMILES (ref. [16])	902	38.63	41.20
Atom-in-SMILES	727	46.32	47.62

In Table 4, top-1 string exact and Tanimoto exact accuracy are listed for various tokenization schemes along with the number of predicted SMILES with repeated tokens, $\text {rep-l}|_{P} - \text {rep-l}|_{GT} \ge 2$, where P and GT refer to prediction and ground truth. We observed performance gains using the AIS tokenization, outperforming the baseline by 4.3% in string exacts and 2.9% in T$_{\textrm{c}}$ exacts. Our methodology, having the fewest single-token repeats, alleviated the repetition problem by approximately 10% compared to the atom-wise tokenization scheme. DeepSMILES exhibited the worst degenerate repetition among all tokenization schemes, but its overall accuracy in predicting retrosynthesis was 3.5% lower than the baseline on average. Regardless of the repetition rate, SELFIES showed lower retrosynthesis prediction accuracy than the baseline of SMILES atom-wise tokenization. The overall performance of SmilesPE was about only half of the baseline. This clearly demonstrates that the pseudo-substructures obtained with SmilesPE were not sufficient to capture the chemical change with ease.

### Molecular property prediction tasks

The Table 5 provides a comprehensive comparison of various tokenization schemes for molecular property prediction tasks, highlighting the significance of tokenization in this domain. To evaluate the performance of these schemes, we used the MoleculeNet benchmark datasets [58], which includes three regression tasks: ESOL for estimating solubility, FreeSolv for hydration free energies, and Lipophilicity for octanol/water distribution coefficient, logD at pH 7.4 and three binary classification tasks: BBBP for barrier permeability, BACE for predicting binding results for a set of inhibitors of human beta-secretase 1, and HIV for predicting inhibitor activity. We trained random forest models with a 5-fold cross-validation strategy to ensure reproducible results. The tokenized form of the molecules was converted into one-hot encoding, which was used as the input feature representation for training the models. The regression tasks were evaluated using the root mean squared error (RMSE) metric, while receiver operating characteristic area under the curve (ROC-AUC) was used for evaluating the classification tasks.

Our findings indicate that the choice of tokenization scheme has a significant impact on the performance of molecular property prediction models. In terms of regression tasks, the AIS tokenization scheme demonstrated superior performance, achieving the lowest RMSE values on ESOL (0.553), FreeSolv (0.441), and Lipophilicity (0.683) datasets. In terms of classification tasks, AIS performed strongly, with the highest ROC-AUC values achieved on BBBP (0.885), and the second-highest value achieved on BACE (0.835) and HIV (0.729) datasets. In Table 5, the performances of SMILES, DeepSMILES, and SELFIES schemes were found to be comparable across all the conducted tasks, with SELFIES exhibiting a slightly more favorable performance than SMILES. However, SmilesPE exhibited inconsistent results compared to other tokenization schemes. While it performed the best on the HIV and BACE datasets and performed well on the BBBP and Lipophilicity datasets, its performance was not consistent across all datasets (worst in ESOL and FreeSolv). This inconsistency could be attributed to the limited vocabulary size of SmilesPE, resulting in an inaccurate representation of molecular substructures. Moreover, the representation of molecules as sequences of pseudo-words in SmilesPE may not be suitable for certain types of molecules, leading to poorer performance on specific datasets.

**Table 5:** Performance analysis of tokenization schemes for molecular property prediction using MoleculeNet benchmark suite

	SMILES	DeepSMILES	SELFIES	SmilesPE	AIS
Regression Datasets: **RMSE**					
ESOL	0.628	0.631	0.675	0.689	**0.553**
FreeSolv	0.545	0.544	0.564	0.761	**0.441**
Lip	0.924	0.895	0.938	0.800	**0.683**
Classification Datasets: **ROC-AUC**					
BBBP	0.758	0.777	0.799	0.847	**0.885**
BACE	0.740	0.774	0.746	**0.837**	0.835
HIV	0.649	0.648	0.653	**0.739**	0.729

Comparison of Random Forest regression and classification models with 5-Fold Cross-Validation. Bold emphasis  denotes the highest performing approach

### AIS as a fingerprint

**Fig. 7:**
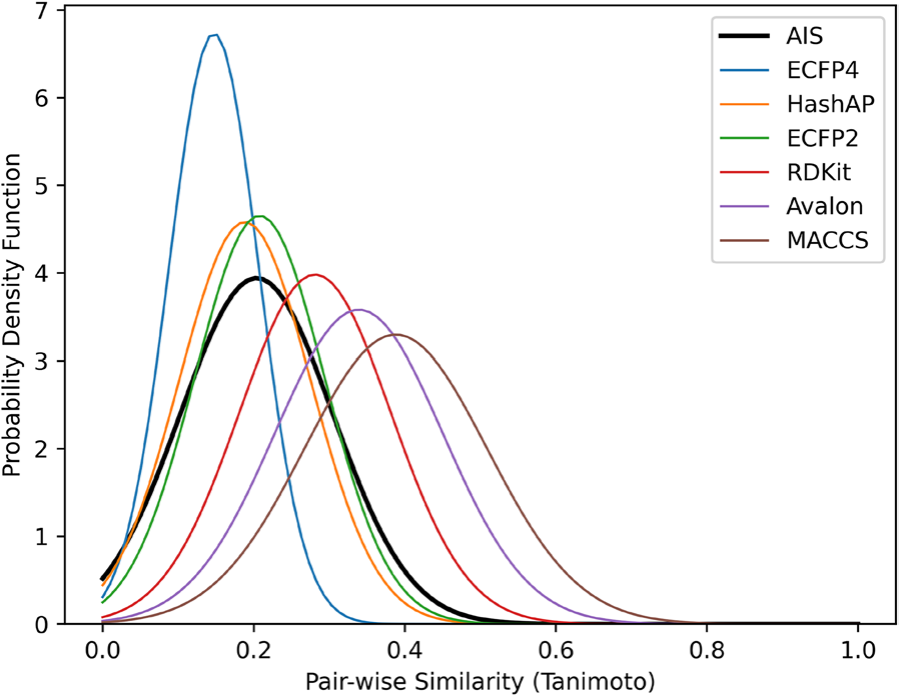
Fingerprint nature of AIS. Pairwise similarity scores of 1 million pairs of molecules are computed for the commonly used structural fingerprints and their probability density functions are plotted. The Tanimoto coefficient is used to measure similarity scores

Due to the compositional nature of the AIS tokenization scheme, it can be readily converted to a fingerprint by simply removing non-atomic tokens. We considered the frequency of each AIS token from a string as its fingerprint form. This is conceptually identical to counting the frequency of each distinct fragment during ECFP generation [50]. It should be noted that computing the molecular similarity with AIS does not require any hashing function. Thus, converting an AIS string to its fingerprint form requires much less computation than other fingerprint methods using hash functions. Based on this definition, we calculated the Tanimoto similarities of 2 million pairs generated by pairwise combination of 2000 randomly chosen ChEMBL molecules using the AIS fingerprint and other widely used fingerprint schemes, and their probability densities are compared (Fig. 7). The most probable similarity between a random pair of molecules using the AIS fingerprint is 0.21. This is similar to those of HashAP and ECFP2 and lower than those of RDKit, Avalon, and MACCS. This indicates that the AIS fingerprint has better resolution power than MACCS, Avalon, and RDKit, and comparable to ECFP2 and HashAP.

This similarity between AIS and its fingerprint form may enhance the learning process of various chemical language models. In general, the loss functions of chemical translation and generation models are assessed through a token-wise comparison. However, few errors in a SMILES string may lead to an invalid or substantially different molecule. Consequently, the loss value and molecular similarities may not be closely correlated. On the contrary, AIS strings with a few token errors represent similar molecules because of the fingerprint-like nature of AIS. Thus, loss values and dissimilarities of molecules due to token errors are more closely correlated with AIS than SMILES.

In a recent study, we established that fingerprint representations, such as ECFP2, ECFP4, and atom environments, can be transformed back into their corresponding SMILES strings with minimal ambiguity [44]. This suggests that fingerprint representations can serve as valuable and informative stand-alone representations. Employing fingerprints as input representations simplifies the application of diverse AI models to chemistry, as bit vectors or straightforward token counts are more manageable than character sequences and can be effortlessly integrated with numerous existing algorithms. We contend that the strong resemblance between AIS strings and their fingerprint counterparts holds significant potential for further development in this domain.

## Conclusion

This study demonstrated that tokenization has a significant impact on the final prediction quality. We introduced atom-in-SMILES (AIS) tokenization as a proper and meaningful custom tokenization scheme to improve the prediction quality in sequence prediction tasks achieving gains of up to 10.7% in equivalent SMILES mapping and 4.3% in a retrosynthetic prediction task. AIS outperforms other tokenization methods in molecular property prediction tasks and aligns more closely with chemical perspectives.

We investigated the resolution of the fingerprint aspect of AIS, revealing that it encompasses all essential information for seamless bidirectional transitions between SMILES and fingerprint representations, ensuring practical implementation. The study addressed the repetition issue in molecular predictions, akin to natural language, which impeded the quality of predicted molecules. The AIS tokenization scheme considerably diminished obstacles in repetitive loops (by around 10%) in the predicted SMILES. As far as we are aware, no prior research has examined token degeneration in AI-driven chemical applications. The AIS tokenization method can be employed by the broader community to deliver chemically precise and customized tokens for molecular prediction, property prediction, and generative models.

## Data Availability

The source code of this work is available via GitHub repo: https://github.com/snu-lcbc/atom-in-SMILES
