# Correction: Improving the quality of chemical language model outcomes with atom-in-SMILES tokenization

**DOI:** 10.1186/s13321-023-00740-w

**Published:** 2023-07-31

**Authors:** Umit V. Ucak, Islambek Ashyrmamatov, Juyong Lee

**Affiliations:** 1 Department of Molecular Medicine and Biopharmaceutical Sciences, Graduate School of Convergence Science and Technology, Seoul National University, Seoul, Republic of Korea; 2 College of Pharmacy, Seoul National University, Seoul, Republic of Korea; 3 Research Institute of Pharmaceutical Science, Seoul National University, Seoul, Republic of Korea

**Correction: Journal of Cheminformatics (2023) 15:55** 10.1186/s13321-023-00725-9

Following publication of the original article [1], the authors requested to correct the funding number NRF-2019M3E5D4066898 to NRF-2022M3E5F3081268.

**Funding**

This work was supported by the National Research Foundation of Korea (NRF) grants funded by the Korean government (MSIT) (Nos. NRF-2022M3E5F3081268, NRF-2020M3A9G7103933, and NRF2022R1C1C1005080). This research was supported by the BK21FOUR Program of the National Research Foundation of Korea (NRF) funded by the Ministry of Education (5120200513755). This work was also supported by the Korea Environment Industry & Technology Institute (KEITI) through the Technology Development Project for Safety Management of Household Chemical Products, funded by the Korea Ministry of Environment (MOE) (KEITI:2020002960002 and NTIS:1485017120).

The original article [1] has been corrected.
